# The α-Gal Syndrome and Potential Mechanisms

**DOI:** 10.3389/falgy.2021.783279

**Published:** 2021-12-16

**Authors:** Patricia Román-Carrasco, Wolfgang Hemmer, Alejandro Cabezas-Cruz, Adnan Hodžić, José de la Fuente, Ines Swoboda

**Affiliations:** ^1^Molecular Biotechnology Section, FH Campus Wien, University of Applied Sciences, Vienna, Austria; ^2^FAZ-Floridsdorf Allergy Center, Vienna, Austria; ^3^Anses, INRAE, Ecole Nationale Vétérinaire d'Alfort, UMR BIPAR, Laboratoire de Santé Animale, Maisons-Alfort, France; ^4^Department of Pathobiology, Institute of Parasitology, University of Veterinary Medicine Vienna, Vienna, Austria; ^5^SaBio, Instituto de Investigación de Recursos Cinegéticos, IREC-CSIC-UCLM-JCCM, Ciudad Real, Spain; ^6^Department of Veterinary Pathobiology, Center for Veterinary Health Sciences, Oklahoma State University, Stillwater, OK, United States

**Keywords:** α-Gal syndrome, red meat allergy, glycoproteins, glycolipids, tick bites, carbohydrates, α-Gal antigen

## Abstract

The α-Gal syndrome is a complex allergic disease characterized by the development of specific IgE antibodies against the carbohydrate galactose-α-1,3-galactose (α-Gal), an oligosaccharide present in cells and tissues of non-primate mammals. Individuals with IgE antibodies to α-Gal suffer from a delayed form of anaphylaxis following red meat consumption. There are several features that make the α-Gal syndrome such a unique allergic disease and distinguish it from other food allergies: (1) symptoms causing IgE antibodies are directed against a carbohydrate moiety, (2) the unusual delay between the consumption of the food and the onset of the symptoms, and (3) the fact that primary sensitization to α-Gal occurs *via* tick bites. This review takes a closer look at the immune response against α-Gal, in healthy and in α-Gal allergic individuals. Furthermore, the similarities and differences between immune response against α-Gal and against the other important glycan moieties associated with allergies, namely cross-reactive carbohydrate determinants (CCDs), are discussed. Then different mechanisms are discussed that could contribute to the delayed onset of symptoms after consumption of mammalian meat. Moreover, our current knowledge on the role of tick bites in the sensitization process is summarized. The tick saliva has been shown to contain proteins carrying α-Gal, but also bioactive molecules, such as prostaglandin E2, which is capable of stimulating an increased expression of anti-inflammatory cytokines while promoting a decrease in the production of proinflammatory mediators. Together these components might promote Th2-related immunity and trigger a class switch to IgE antibodies directed against the oligosaccharide α-Gal. The review also points to open research questions that remain to be answered and proposes future research directions, which will help to get a better understanding and lead to a better management of the disease.

**Graphical Abstract:**
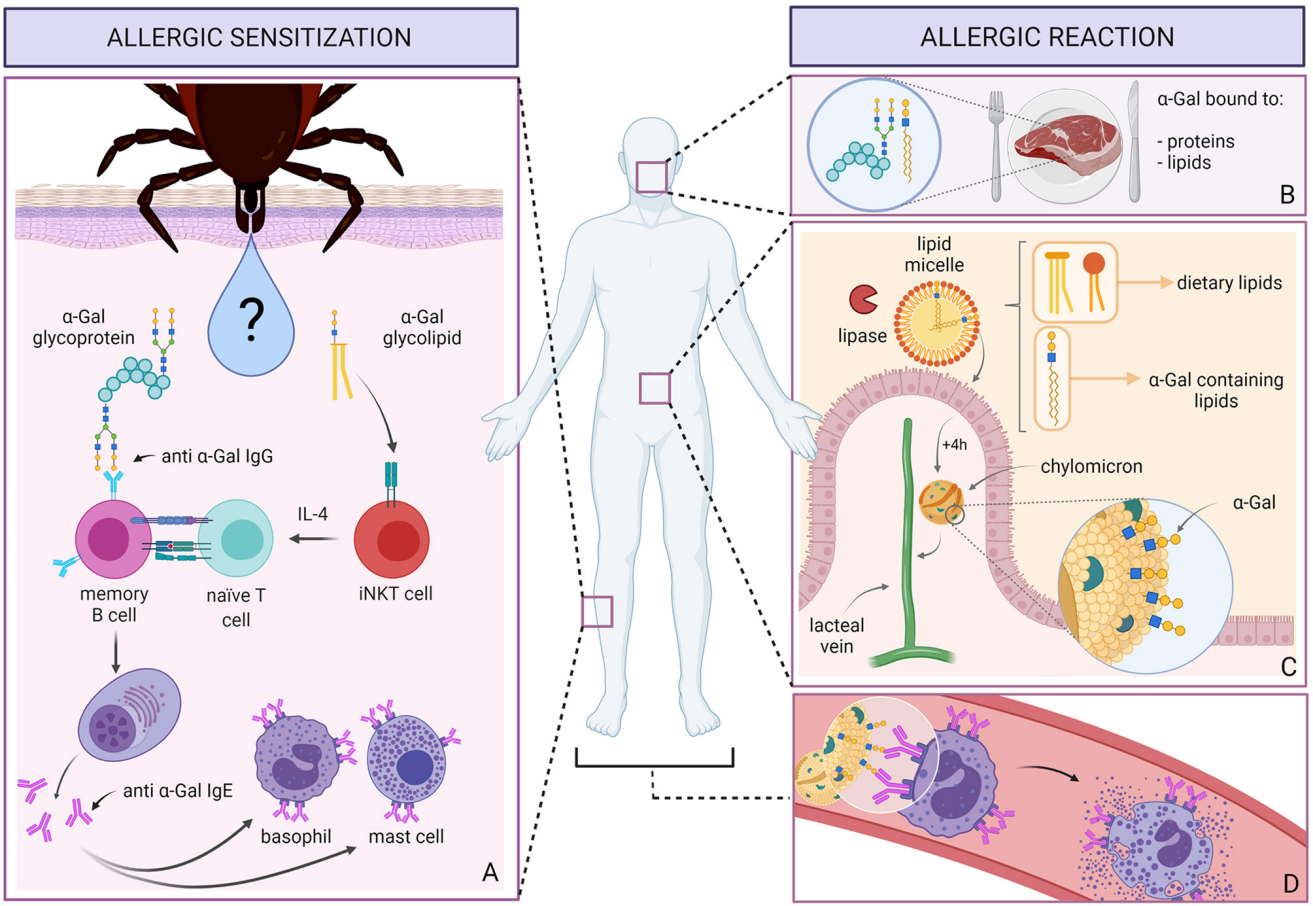
Allergic sensitization to α-Gal **(A)** occurs after repeated tick bites. Among immunomodulatory molecules, tick saliva contains proteins glycosylated with α-Gal and most likely also α-Gal containing glycolipids. α-Gal on proteins is recognized by memory B cells expressing anti-α-Gal B cell receptors. In contrast, glycolipids might be recognized by iNKT cells, which can then produce IL-4. B cells process the glycoproteins and present the peptides to naT cells. In the IL-4 rich milieu, T cells then induce the class switch recombination of B cells leading to the production of IgE to α-Gal, which then bind to basophils and mast cells. When sensitized individuals ingest red meat containing α-Gal bound to proteins and lipids **(B)**, these glycolipids are incorporated into lipid micelles **(C)**. Pancreatic lipase, an enzyme active at water-lipid interfaces, hydrolyzes the triglycerides inside the micelle into free fatty acids, mono- and diglycerides, which are absorbed by enterocytes. About 4 h later, processed lipids, packed in chylomicrons and presumably coated with α-Gal molecules, are released into the lymph via the lacteal vein. When chylomicrons reach the blood stream and tissues **(D)**, they encounter basophils and mast cells coated with IgE antibodies to α-Gal. The α-Gal moieties displayed on the surface of chylomicrons can then cause the cross-linking of IgEs and the subsequent degranulation of basophils and mast cells leading to a systemic allergic reaction.

## Introduction

The carbohydrate galactose-α-1,3-galactose (α-Gal) has been known for a long time as a barrier for xenotransplantation (1), preventing the transplantation of pig organs into humans. The reason for organ rejection is that humans produce antibodies against the α-Gal oligosaccharide, which is abundantly expressed on glycoproteins and glycolipids in mammalian cells, except in humans, apes and Old-World monkeys. The interaction between these antibodies and the α-Gal epitope activates the complement cascade, which results in destruction of the xenograft (2).

However, a decade ago, α-Gal gained further attention for being the molecule responsible for an uncommon form of food allergy, the so-called α-Gal syndrome, where patients produce IgE antibodies against the carbohydrate α-Gal. These patients develop delayed allergic reactions after consumption of mammalian meat, such as beef, pork, or lamb (3), and immediate allergic responses after intravenous administration of α-Gal-containing drugs, such as the anticancer monoclonal antibody Cetuximab (4). The delayed onset of symptoms about 3–6 h after red meat (5) consumption is indeed remarkable, since IgE-mediated hypersensitivity reactions to foods are usually of rapid onset and symptoms appear within minutes but generally within 2 h after eating the offending food. The delay in the occurrence of symptoms after meat consumption was proven in food challenge studies, whereas it was shown that patients' basophils could be activated immediately upon *in vitro* exposure to α-Gal (5). These findings suggested that the delay in the symptoms is neither caused by an intrinsic property of the carbohydrate nor by a delay in basophil responsiveness, but rather by the time taken by α-Gal molecules from the ingestion until the appearance in the circulation. Owing to the slower digestion and absorption of lipids as compared to proteins, it was suggested that α-Gal bound to lipids, and not to proteins, could be behind the late response of α-Gal allergic patients (6).

Another yet to be answered question about the α-Gal syndrome is why certain individuals produce IgE antibodies against the carbohydrate moiety α-Gal. Immune responses to carbohydrates are thought to occur without T cell help. However, the production of IgE antibodies involves class-switching that requires the input from T cells. Sensitization to α-Gal usually starts in adults or adolescents, affecting often individuals with no atopic disposition. In most cases, patients had consumed red meat without complications for many years prior to the onset of the symptoms. Surprisingly, thorough investigations provided evidence that tick bites trigger the production of IgE antibodies against α-Gal (7). From an evolutionary standpoint it was proposed that allergic responses to tick bites induce ‘allergic klendusity', an allergy-mediated immunity that protects against ticks and tick-borne pathogens (8). Even though the molecules present in the tick saliva and the immune mechanisms leading to the α-Gal syndrome still need to be discovered, these findings revolutionized our knowledge on allergic diseases: it was shown for the first time that an ectoparasite is capable of inducing IgE antibodies against a molecule present in its saliva that can later trigger food allergic reactions.

## Ige Antibodies Against Carbohydrates

For many years, carbohydrates have been regarded as poorly allergenic molecules. Complex polysaccharides were traditionally considered as T cell-independent antigens and therefore, they were thought not to be involved in the development of adaptive immune responses, immunoglobulin class-switching and immunologic memory (9). The capability of sugar moieties to alter the recognition of protein antigens by T cells has been known for a long time (10). However, it was only recently that the ability of glycans to modulate innate and adaptive immune responses started to be better understood (11). Research has meanwhile shown that carbohydrates as part of glycoproteins or glycolipids can either represent T cell epitopes themselves or that they can participate in the formation of T cell epitopes (12). Differences in the structure, size, complexity and location of the glycan can influence the binding of a peptide to major histocompatibility (MHC) molecules and thus the recognition of the peptide by T cells (12). In addition, glycosylation may also influence the direction of the immune response and can, for instance, skew the immune system toward a Th2 type response, as in the case of glycans expressed by some helminths (13–15).

As a matter of fact, the existence of IgE antibodies directed against glycans bound to proteins from plants and insects has been known for many years (16). In 1981, Aalberse et al. (16) showed that IgE antibodies from some patients cross-reacted with carbohydrates in the extracts from different unrelated vegetable foods, pollen and with insect venoms. These antigens were therefore named cross-reactive carbohydrate determinants (CCD). Interestingly, tick saliva composition is very similar to the venom of known venomous animals (17, 18). Later, it was shown that the structural basis for the cross-reactivity of patients' IgE antibodies with insect and plant glycoproteins was an oligosaccharide with a core α-1,3 fucose and/or a xylose (19, 20). However, IgE antibodies directed to CCDs showed poor biological activity and apparently lacked the ability to induce allergic reactions (20). This lack of clinical relevance of anti-CCD IgE antibodies was further proven in a study (21) where grass pollen allergic patients with IgE antibodies to CCDs underwent an oral challenge with lactoferrin expressing CCD glycans and results were completely negative (21). Thus, it was concluded that IgE antibodies against CCDs lack clinical relevance. Later, it was also confirmed that CCDs are a main reason for false positive *in vitro* results (22, 23).

More than 25 years after the discovery of CCDs, however, the existence of IgE antibodies directed against another glycan, namely the mammalian oligosaccharide, α-Gal, was reported. Interestingly, IgEs to α-Gal were shown to be involved in the development of anaphylactic reactions (4, 6). First indications came from observations in the Southeast region of the United States, where a high proportion of patients treated with the monoclonal anti-cancer antibody Cetuximab showed severe hypersensitivity reactions to the drug. Chung et al. discovered that these patients had IgE antibodies to Cetuximab and further investigations on the epitope responsible for the IgE reactivity showed that the Fab portion of Cetuximab, glycosylated with a range of sugars, including α-Gal, is recognized by patients' IgE antibodies. The proof that the IgE antibodies were directed against α-Gal came from experiments performed with a Cetuximab variant that lacked α-Gal and did not show IgE reactivity (4). Later, it was seen that these IgE antibodies directed against α-Gal could trigger a new delayed form of food-induced anaphylaxis, in which the onset of symptoms, different to protein food allergies, occurs 3–6 h after eating mammalian meat (24, 25).

## Clinical Characteristics of the α-Gal Syndrome

One of the most characteristic features of the α-Gal syndrome, and the one which also attracted particular attention, is the delayed onset of allergic symptoms after food consumption. Although cases with earlier onset of symptoms have been described (26), in most patients with IgE antibodies against α-Gal the onset of the symptoms occurs 3–6 h after the intake of mammalian meat (27, 28). This delay in reactions differs significantly from the onset of symptoms caused by protein food allergens, which typically starts within 1 h, often within minutes, following ingestion of the allergenic food. However, food allergic reactions are only one aspect of the α-Gal syndrome, the other aspect being reactions after infusion or injection of drugs and other medical products with α-Gal containing mammalian ingredients. The best studied reactions are immediate anaphylactic reactions following first-time intravenous administration of cetuximab, a chimeric mouse-human monoclonal IgG antibody against the epidermal growth factor receptor (EGFR), which is applied in patients with metastatic colorectal cancer (29). These reactions can be rapid in onset and even immediate (29).

Allergies to food proteins manifest in several clinical conditions and cause symptoms in the gastrointestinal tract (e.g., oral itch, laryngeal edema, nausea, vomiting, cramps, and diarrhea), the skin (e.g., urticaria, angioedema, and atopic dermatitis) and the respiratory tract (e.g., rhinitis, dyspnea), and they are also considered as an important cause of fatal anaphylactic reactions (30). Among adults, the most common clinical manifestation of protein-based food allergy is the so called oral allergy syndrome (OAS) (30). In α-Gal allergic patients, the most observed symptoms after red meat consumption involve cutaneous manifestations, such as itching, erythema, urticaria and angioedema. Often, reactions also include severe and even life threatening anaphylaxis (31). These clinical manifestations can be accompanied by gastrointestinal symptoms. Although abdominal pain alone, without concurrent hives or other skin reactions has been reported, these symptoms are described to be mainly subjective (26, 32). Thus, isolated gastrointestinal symptoms appear to be rather rare and mouth itching or swelling are also uncommon (33, 34).

Allergic reactions to α-Gal present in red meat or in drugs of mammalian origin usually start in adult life, and only rarely during childhood (35). Furthermore, atopic disposition is not associated with a higher risk of developing the α-Gal syndrome (33). These features are clearly in contrast to IgE-mediated allergies to food proteins, which develop in genetically predisposed individuals often early in life, and which show higher incidences in children (prevalence of 6–8%) than in adults (2–5%) (36). Some food allergies, such as milk, egg and peanut allergy, predominantly affect children and frequently resolve in childhood or adolescence, and patients can later consume these foods without experiencing any allergic reactions. In contrast, α-Gal allergic patients can often consume red meat without complications for many years prior to the onset of symptoms. It is then the bite of a tick that causes sensitization to α-Gal and the development of the α-Gal syndrome. However, a number of recent studies showed that the type of mammalian meat, as well as several co-factors are relevant in determining whether symptoms occur and how severe they are. For instance, consumption of fattier forms of meat and of mammalian innards, such as pork kidney, which are known to contain more α-Gal epitopes than muscle meat, can lead to more severe and rapid reactions (37). Furthermore, alcohol consumption and physical exercise and the use of certain medications (e.g., Nonsteroidal anti-inflammatory drugs—NSAIDs), are known to lower reaction thresholds to food allergens and may lead to more severe clinical presentation, or faster onset of the reactions (38). Physical exercise and alcohol consumption increased the severity of symptoms in 5 and 10% of food allergic patients respectively (38). These co-factors appear to affect also the course of allergic reactions to α-Gal (39). It is thought that the co-factors increase the gastrointestinal permeability (38), which might affect the absorption of α-Gal, but also the release of histamine (38, 39).

## Diagnosis of α-Gal Allergic Patients

Food allergy diagnosis can be challenging since patient history is frequently unreliable and *in vivo* skin prick tests and *in vitro* determination of specific IgE levels may prove sensitization but not clinical hypersensitivity. The diagnosis of the α-Gal syndrome can be even more difficult, because the use of skin prick tests appears to be unreliable for diagnosis of α-Gal allergic patients. α-Gal allergic patients produce no or only small wheal and flare reactions of 2–4 mm in diameter, when prick tests are performed with commercial extracts of beef, pork, or lamb. Such weak reactions may be interpreted as negative and can lead to incorrect recommendations to patients. In contrast, skin prick testing with fresh meat extracts or testing meat extracts intradermally can induce strong positive results (3, 33), but this approach is not feasible for routine practice.

An explanation for the weak reactions obtained in prick tests could be that the concentration of α-Gal epitopes in commercial protein extracts used for skin prick tests might be rather low. In the case of the meat protein extracts used in the prick tests, the protein concentration may be known, but not the abundance and distribution of α-Gal. If many of the proteins in the extract are not highly decorated with α-Gal moieties, it might not be possible to cross-link IgE-antibodies on the surface of a sufficient amount of mast cells to elicit a response that is considered positive. Interestingly, highest sensitivities were observed when the tests were performed with native kidney pork in the form of prick-to-prick tests (39). Pork kidney also elicits symptoms more consistently than muscle meat, which can be explained by the fact that pork kidney appears to have higher concentrations of α-Gal epitopes (37).

Interestingly, correlations between the abundance of sugar moieties in skin prick test solutions and the appearance of positive skin reactions have also been described in CCD-positive individuals, where the effects of the CCD-carrying glycoproteins ascorbic acid oxidase, phospholipase A2 and horseradish peroxidase (HRP) were compared. When tested with phospholipase A2 or ascorbate oxidase, patients did not show any skin reaction. However, patients did react, although weakly, to horseradish peroxidase (40). Whereas phospholipase A2 and ascorbate oxidase have only two N-glycosylation sites, HRP has up to eight (41), which would result in a higher number of glycan moieties in the HRP extract (41), making the cross-linking of IgE antibodies on mast cells more likely. Comparably, if the proteins in meat extracts are not highly N-glycosylated with α-Gal-containing sugars, they would not be able to elicit mast cell degranulation during skin prick test. In contrast, during prick-to-prick tests, especially if these are carried out using pork kidney, not only α-Gal containing proteins, but also lipids, are inoculated. The higher concentration of α-Gal molecules could be, therefore, responsible for the activation of the mast cells.

Oral food challenge, regarded as the gold standard for food allergy diagnosis, may also represent the gold standard for the diagnosis of the α-Gal syndrome, but it bears the risk of inducing severe, potentially life-threatening anaphylactic reactions. Moreover, oral food challenge is limited in practice to a few specialized centers or clinical trials because of insufficient opportunities for food provocations in a predominantly adult patient population in routine settings (32, 42).

Currently, *in vitro* determination of α-Gal specific IgE antibodies in patients' sera using the ImmunoCAP assay represents the most reliable method for the diagnosis of α-Gal sensitization (33). However, the use of this assay in a population of German forest service employees and hunters who are highly exposed to ticks, showed that elevated IgE levels >0.1 kUA/L against α-Gal were present in 35% of this group, but also in 15% of an age-matched control group from the general population (43). Thus, sIgE to α-Gal did not necessarily correlate with clinical meat allergy, but α-Gal serum IgE positivity was associated with recent tick bites. In fact, only 5% of α-Gal IgE-positive subjects suffered from a clinical α-Gal syndrome. It was therefore concluded that determination of anti-α-Gal IgE levels cannot distinguish between patients suffering from the α-Gal syndrome and individuals with asymptomatic α-Gal sensitization, showing the limitations of an α-Gal syndrome diagnosis that is only based on anti-α-Gal IgE titer determinations (44). Nevertheless, the *in vitro* test allows identifying individuals at a risk of developing an α-Gal syndrome. Since α-Gal allergic individuals are frequently non-atopic individuals with low total IgE levels, it has been suggested to compare specific anti-α-Gal IgE levels with total IgE levels (26, 33). Anti-α-Gal IgE levels higher than 2% of the total IgE levels would mean a positive diagnosis (33). Moreover, Mehlich et al. (45) suggested to use basophil activation tests for differentiation between α-Gal allergic patients and mere α-Gal sensitized but asymptomatic patients. Their suggestion was based on the observation of higher basophil reactivity and sensitivity in patients with the α-Gal syndrome as compared to sensitized individuals. The limitation of the basophil activation test is that it can only be performed in specialized laboratories.

Instead, it has been suggested that integrative tools, such as algorithm implemented in mobile applications that consider clinical symptoms, risk factors and anti-α-Gal IgE levels, could be applied in the future for easier diagnosis of the α-Gal syndrome in the clinical practice (44).

## The α-Gal Epitope and Antibody Responses to α-Gal

**Figure 1:**
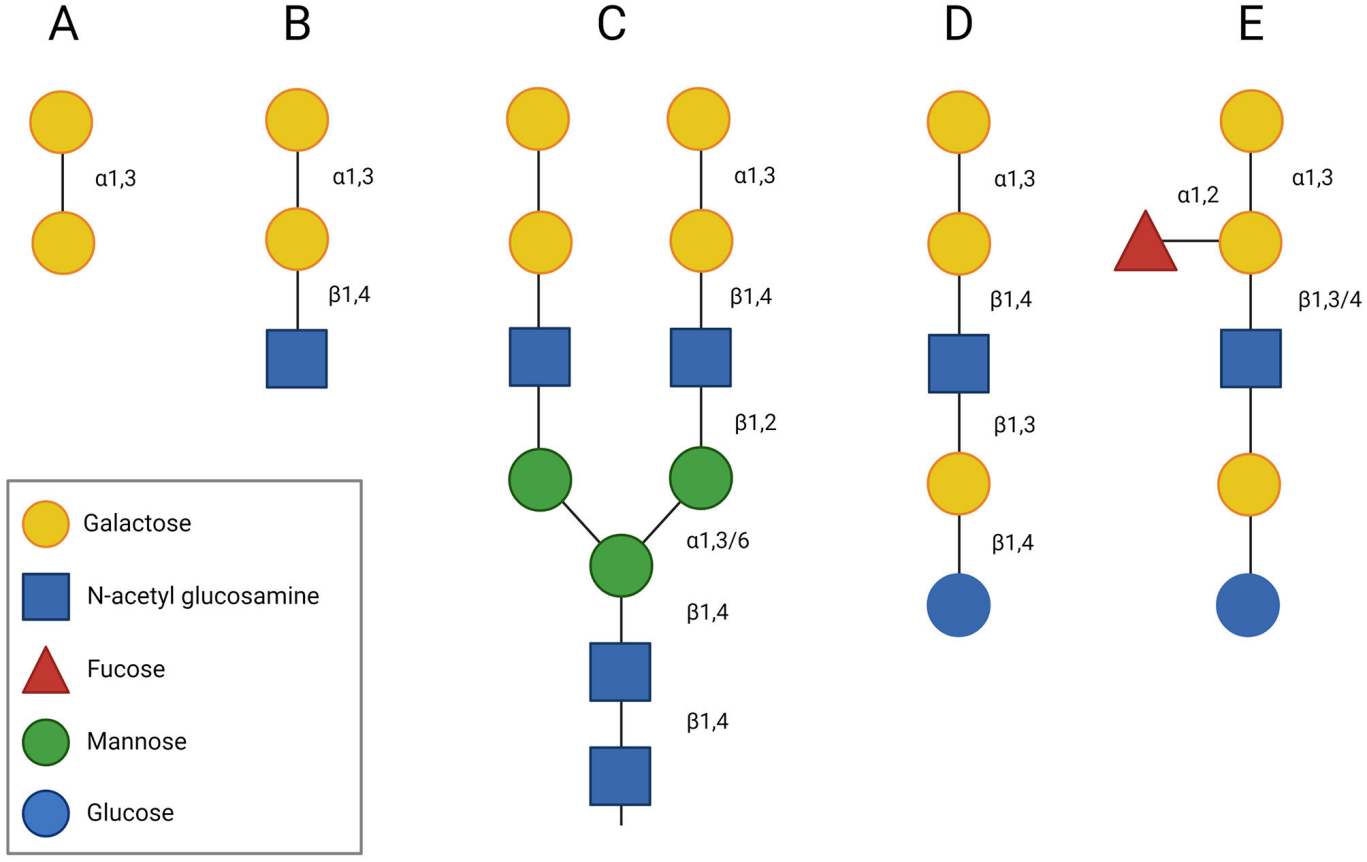
Structure of the α-Gal epitope. The core structure of the α-Gal epitope is the terminal disaccharide galactose-α-1,3-galactose **(A)**, which is often followed by N-acetyl glucosamine (GlcNAc) in the third position forming a trisaccharide (Gal-α-1,3-Gal-β-1,4-GlcNAc) **(B)**. In glycoproteins, the α-Gal epitope can occur as part of a bi-antennary N-glycan **(C)**, whereas in glycolipids it can be part of a pentasaccharide **(D)**, whose structure resembles the structure of the human B blood group antigen **(E)**.

The core structure of the α-Gal epitope is the non-reducing terminal disaccharide galactose-α-1,3-galactose (Gal-α-1,3-Gal) (Figure 1A), which is usually followed by N-acetylglucosamine (GlcNAc) in the third position (Figure 1B). The resulting trisaccharide, the α-Gal epitope Gal-α-1,3-Gal-β-1,4-GlcNAc, is expressed on glycoproteins (Figure 1C) and glycolipids (Figure 1D) of mammals, except Old World monkeys, apes, and humans. It is believed that an evolutionary event, about 28 million years ago, led to the inactivation of the gene for α-1,3-galactosyltransferase (α1,3GT), the enzyme responsible for the synthesis of α-Gal, in ancestral Old World primates. In human cells, truncated transcripts of the α1,3GT gene have been detected. However, these mRNAs lack the two catalytic exons and thus, their translation results in an inactive α1,3GT enzyme (46). Instead of expressing the α-Gal epitope on glycoconjugates, Old World monkeys, apes and humans produce antibodies against this oligosaccharide (47). It is estimated that around 1% of circulating antibodies in healthy individuals are directed against α-Gal (48). These antibodies and their interaction with the α-Gal epitope present on organs of mammalian origin (e.g., pig organs) can activate the complement system, resulting in hyperacute reactions in xenotransplantation (1).

Investigations of the classes of anti-α-Gal antibodies revealed the presence of IgG, IgM, and IgA antibodies in human serum, whereas the IgA isotype clearly dominated in human secretions, such as the saliva, colostrum, milk, bile, and vaginal washings (49). Anti-α-Gal IgA antibodies seem to represent a significant proportion of the total secretory immunoglobulins and they have been shown to belong to both subclasses, IgA1 and IgA2 (49).

Analysis of antibody binding to the α-Gal epitope showed that the terminal Gal-α-1,3-Gal disaccharide structure is sufficient for antibody recognition, but that sugar residues following the terminal disaccharide can modify the specificity of the binding. Milland et al. assessed the reactivity of different monoclonal antibodies (mAb) and found that these mAb can differentiate between glucose (Glc) and N-acetylglucosamine (GlcNAc) present in the third position of the α-Gal epitope (50). Analysis of human natural IgG and IgM antibody responses to α-Gal showed heterogeneity in the antibody populations (51). Furthermore, these analyses revealed that all human anti-α-Gal antibodies recognized the terminal Gal-α-1,3-Gal disaccharide, but, as Milland et al. (50) observed using monoclonal antibodies, the addition of further carbohydrates at the reducing terminus increased the efficiency of the binding. Inhibition studies performed with human serum and α-Gal oligosaccharides showed that α-Gal tri-, tetra-, and pentasaccharides were more efficient in blocking anti-α-Gal antibodies than the disaccharide only (52). Overall, the pool of natural human anti-α-Gal antibodies appears to have low specificity and antibodies might also bind to carbohydrates structurally different from α-Gal (53). This is also supported by the observation that patients with chronic Chagas disease develop highly specific lytic antibodies against O-linked α-Gal of the *Trypanosoma cruzi* trypomastigote F2/3 antigen complex. These antibodies show 100 times higher affinity for α-Gal than the natural anti-α-Gal antibodies present in the serum of healthy individuals (54).

Interestingly, the chemical structure of the α-Gal epitope is very similar to the blood group B antigen (Figure 1E). Both antigens share the two terminal galactoses connected with an α-1,3 bond. They differ only in a fucose joined to one of the terminal galactoses by an α-1,2-glycosidic linkage in case of the B antigen. Galili et al. (55) demonstrated that some of the anti-α-Gal IgG antibodies can also recognize the blood group B antigen and McMorrow et al. (56) showed that blood group B antigen-expressing individuals (blood groups B and AB) have a lower overall level of α-Gal IgG antibody reactivity as compared to non-B antigen-expressing individuals (blood groups 0 and A). This clearly showed that there is a correlation between antibody responses to α-Gal and to the blood group B antigen (57).

Characterization of the IgE antibody response to α-Gal revealed that also IgE antibodies recognize the Gal-α-1,3-Gal disaccharide structure (Figure 1A) and that IgE antibodies bind to both terminal galactose molecules of the α-Gal epitope (58). Addition of GlcNAc in the third position (Figure 1B) resulted in most cases in an increased IgE reactivity to the α-Gal epitope (59). Besides, it has been observed that some anti-α-Gal IgE antibodies from red meat-allergic patients can also bind to the blood group B antigen (60). Further investigations then showed that IgE antibodies to α-Gal are significantly less abundant in individuals with blood groups B or AB and that α-Gal allergic subjects usually belong to the B antigen negative blood groups (A and O), and only rarely to the B antigen positive blood groups (B and AB) (60, 61).

In addition to the correlation with the blood group B antigen, antibody responses to α-Gal are highly variable among individuals and interestingly IgE and IgG antibody responses to α-Gal are apparently associated. Significantly higher titers of anti-α-Gal IgG1 antibodies can be found in α-Gal allergic patients with IgE antibodies to α-Gal than in healthy individuals (56, 60, 62). Our group further compared the anti-α-Gal IgG subclass response in α-Gal allergic individuals with the anti-α-Gal response in two other groups of allergic individuals, patients with allergy to a food protein and subjects with IgE antibodies to CCDs (63). Both patient groups, those allergic to α-Gal and those with IgEs to CCDs, had higher titers of anti-α-Gal IgG2, but specially of anti-α-Gal IgG1 antibodies than the group of individuals allergic to the food protein. Remarkably, CCD allergic patients, also had elevated IgG1 levels (but not IgG2 antibodies) against CCDs. Based on these results, it seems likely that certain individuals are more prone to develop antibodies against carbohydrates than others (63). The reasons for these differences in the humoral responses to carbohydrates and specifically to α-Gal are not yet known. It could be envisaged that inter-individual variations in the composition of the microbiota present in the gut of every person might be responsible for the development of different anti-carbohydrate antibody responses.

The presence of IgE antibodies directed against α-Gal is associated with harmful allergic reactions to mammalian meat and to α-Gal containing drugs (6). However, antibody responses to α-Gal can also have positive effects. In malaria endemic regions, it was observed that IgM antibody responses to α-Gal may prevent infection by *Plasmodium falciparum* (64, 65). Comparably, anti-α-Gal antibodies have shown to confer protection against Chagas disease (66, 67) and leishmaniasis (68, 69) and experiments in α-Gal knock-out mice confirmed that gut colonization by *E. coli* O86:B7, a human gut bacterium expressing α-Gal, was followed by the development of IgM antibodies to α-Gal. These antibodies further conferred protection against malaria transmission to the mice (64). Interestingly, it was also shown that anti-α-Gal IgA antibodies from human colostrum can bind to several gram-negative commensal bacteria and were able to inhibit the adhesion of *Neisseria meningitidis* to human buccal cells (49). These findings suggested that the secretory anti-α-Gal IgA antibodies could play a protective role at mucosal surfaces.

## Occurrence of α-Gal

Among vertebrates, the α-Gal epitope, Gal-α-1,3-Gal-β-1,4-GlcNAc, is only expressed on glycoproteins and glycolipids of mammals, whereas fish, amphibians, reptiles, and birds do not produce this glycan moiety (70). Placental mammals, such as mice, cats, dogs, horses, cows, pigs, bats, New World monkeys or dolphins, as well as marsupials, such as opossums and kangaroos, all produce large amounts of α-Gal on all different kinds of cell types, such as fibroblasts, endothelial cell, epithelial cells, muscle cells and lymphoid cells (70). However, as mentioned before, there are a few mammals, namely Old-World monkeys, apes and humans, that lack the enzymatic machinery to synthesize α-Gal. Humans and animals (e.g., birds, fish) lacking α-Gal can produce antibodies against α-Gal (71). It is believed, that the production of these α-Gal specific antibodies is a response to the continuous antigenic stimulation with α-Gal epitopes bared by some bacteria in the gut (54, 72). Indeed, glycans play an important role in the interaction between hosts and pathogens (73–75) and in 1988, Galili et al. were the first to describe that beside mammals also bacteria can express the α-Gal epitope. It was shown that various commensal *Escherichia coli, Klebsiella*, and *Salmonella* strains, several of them isolated from humans stool samples, express α-Gal either linked to glycoproteins of the bacterial capsule and cell wall or to lipopolysaccharides (LPS) (54). Meanwhile, it has been shown that also other bacteria, like *Serratia* and *Mycobacteria* strains, the tick-borne bacteria *Borrelia burgdorferi* and *Anaplasma phagocytophilum* (76), the helminth *Schistosoma mansoni* (77), the fungus *Aspergillus fumigatus* (77) as well as some pathogenic protozoan parasites, such as *Trypanosoma, Plasmodium* spp., or *Leishmania* spp., which cause Chagas disease, malaria and leishmaniasis, respectively, have α-Gal moieties on their surface (64, 78, 79). However, in case of the *Plasmodium falciparum* sporozoites, the origin of the α-Gal moieties is not yet clear, since *Plasmodium falciparum* lacks some glycosyltransferases genes required for the synthesis of N-glycans. Interestingly, galactosyltransferase genes with high homology to those recently described in ticks (80) are widespread among insects including the mosquitoes *Aedes aegypti and Anopheles gambiae* (81), and there is some evidence that α-Gal found on the surface of *Plasmodium* oocytes and sporocytes actually stems from the vector (82). As antibodies against α-Gal have been shown to inhibit *Plasmodium* growth, it has been speculated that increased resistance to malarial infection due to preformed natural anti-α-Gal antibodies may have been a major reason for the evolutionary loss of α1,3GT activity in the Catarrhini (83). Nevertheless, despite the presence of homologous genes of enzymes with α1,3GT activity, mosquito bites or saliva do not seem to elicit effective immune responses against *Plasmodium* sporozoites in α1,3GT knockout mice, that lack the ability to synthesize α-Gal (84).

Furthermore, α-Gal can also be linked to glycoproteins present in the envelop of zoonotic viruses, if these viruses replicate in organisms showing α1,3GT activity, such as non-primate mammalians, ticks and insects. Transmission of such viruses to humans may be caused by bites, or by aerosolized, fecal, and urinary secretions from these mammals or by consumption of these mammals (85). There is evidence that anti-α-Gal antibodies then protect humans against the zoonotic viruses by destroying and neutralizing them and by targeting them for uptake by antigen-presenting cells (85). α-Gal has been found also on envelop proteins of arboviruses such as Eastern Equine Encephalitis virus and Sindbis virus cultured in mammalian cell lines (86, 87). Assuming that α-galactosylation also occurs in the respective insect vectors, many different bloodsucking insects (e.g., mosquitoes, black flies, horseflies, ceratopogonids, sand flies, and kissing bugs), which transmit a variety of arboviruses with different pathogenicity, may therefore inoculate α-Gal into the human skin during biting. It is yet unknown if insect saliva itself harbors α-Gal epitopes, as assumed for ticks. Considering that, similar to ticks, virtually all blood-feeding insects regularly elicit a saliva-specific immune response in the host, commonly characterized by Th2 polarization and induction of specific IgE antibodies (88), the potential role of blood-feeding insects in the induction of α-Gal-specific IgE antibodies might warrant further studies.

## Presence of α-Gal in Food

The oligosaccharide α-Gal is expressed in different mammalian tissues. Besides mammalian muscle, α-Gal can be also found in organs such as kidney, liver, spleen, or heart of mammals (89). Quite some effort went into the identification of α-Gal carrying proteins in beef and pork and it was shown that α-Gal was bound to several proteins in raw and in cooked beef (90), among them α-enolase, ß-enolase, laminin and collagen (91). In pork kidney (92), aminopeptidase N and angiotensin-I-converting enzyme were identified as α-Gal-carrying proteins.

Interestingly, in case of muscle tissue, patients appear to have more severe reactions after eating fattier meats, whereas they tolerate leaner cuts (33), which suggests that glycolipids carrying α-Gal might be involved in the induction of allergic reactions. Therefore, α-Gal allergic patients are also advised to avoid meat broth and bouillon or food containing mammalian fat, which is for instance used for the preparation of certain sauces, pastries, mashed potatoes, but also vegetable dishes and desserts. Owing to the presence of glycolipids in pork small intestine, certain chicken sausages, where minced chicken meat is stuffed in pig tripe, should also be avoided, because they might also trigger symptoms in patients suffering from the α-Gal syndrome (33). Even though innards have a lower total fat content than muscle tissue, ingestion of only small amounts (1–2 g) of pork kidney can already trigger allergic reactions during oral challenges (37) and eventually even patients who do not react to meat muscle show symptoms after eating organs, such as pork kidney (93). However, innards, like kidney, small intestine, liver, spleen, salivary glands and heart, are generally richer in cholesterol, and high levels of α-Gal expressing glycolipids have been detected in different pig organs (37, 89), which clearly suggests that α-Gal glycolipids might be responsible for the high allergenicity of innards.

**Figure 2:**
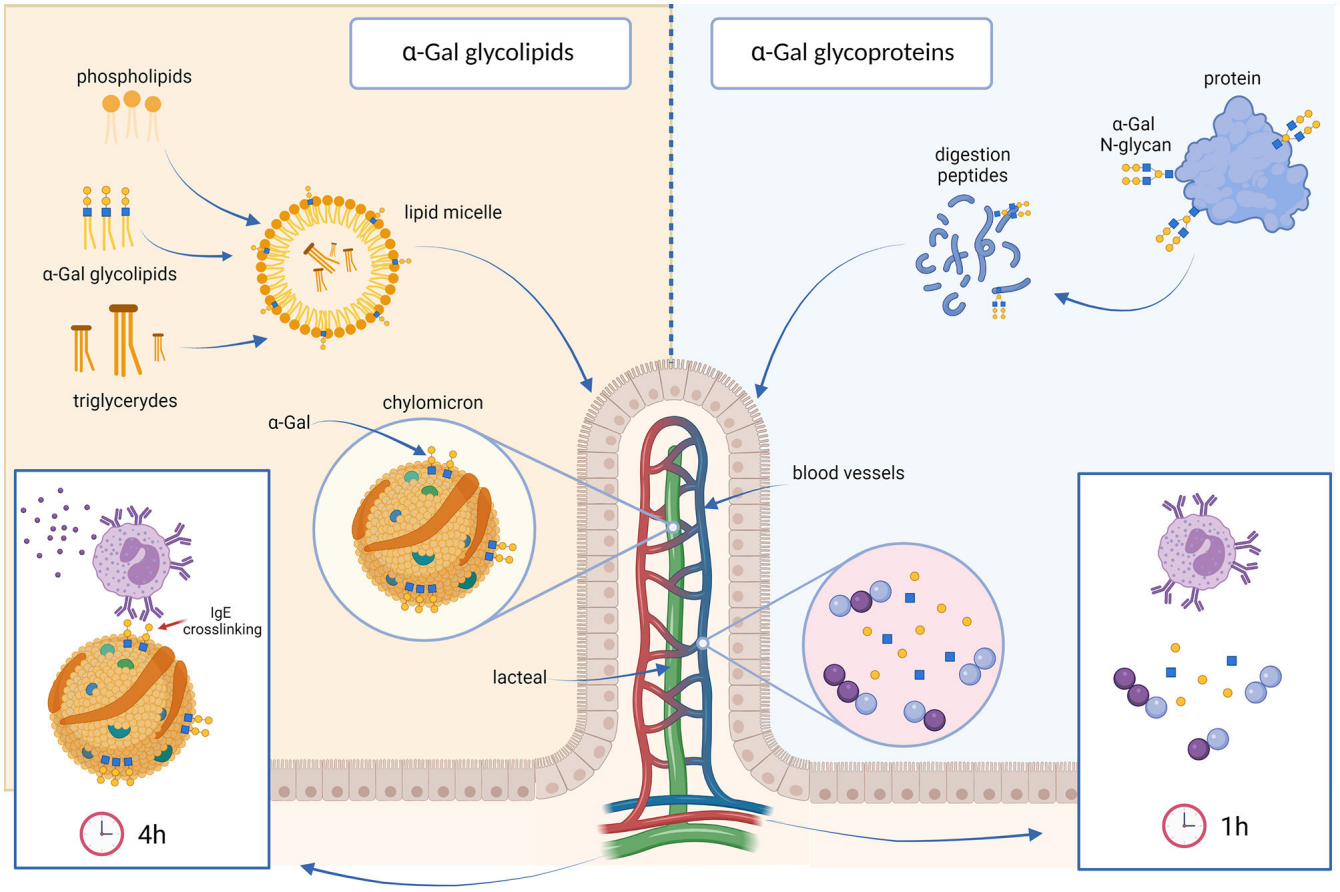
The glycolipid hypothesis. Dietary lipids are broken down into small droplets, which are coated by bile salts and phospholipids to form lipid micelles **(left)** that are absorbed by the enterocytes. α-Gal glycolipids are most likely incorporated into these lipid micelles. In the enterocytes, lipids are packaged into lipoprotein particles, called chylomicrons. In this form, lipids are transported into the lymph via the lacteal vein, reaching the blood stream in the subclavian vein 3 to 4 h after a meal. α-Gal molecules would be exposed on the surface of the chylomicrons, allowing the cross-linking of IgE antibodies directed against α-Gal. This could explain the delay in the allergic reactions of α-Gal allergic individuals after eating red meat. In contrast, glycoproteins carrying α-Gal are digested into small peptides and α-Gal bound to them into monosaccharides. The postprandial peak of these digestion products, that would be uncapable of binding or cross-linking anti-α-Gal antibodies, occurs approximately 1 or 2 h after ingestion **(right)**.

As discussed in detail in the next chapter, the binding of α-Gal to glycolipids instead of glycoproteins might also provide an explanation for the delayed occurrence of symptoms after meat consumption, since the digestion and absorption of lipids takes longer than the digestion of proteins (Figure 2). The content of total glycolipids in beef or pork muscle tissue is rather low (94) and usually accompanied by high amounts of triglycerides. Triglycerides are large, water-insoluble molecules that cluster together in big droplets when they get into a watery environment like the digestive tract. During the digestion process of muscle meat, these large droplets are first broken into smaller droplets and are then enzymatically digested by lipases in the small intestine. From there they are then absorbed by the enterocytes. Due to their insolubility in water, the transport of triglycerides into the circulation also requires special modifications and therefore needs more time. However, in the case of innards, with a lower total fat and triglyceride content but with higher amounts of α-Gal glycolipids, lipid droplets are smaller and absorbed faster (95). This may explain why patients usually experience faster and more severe allergic reactions after the consumption of innards than after eating mammalian muscle meat (37).

Even though collagen was suggested as one of the α-Gal carrying proteins (91), the role of its denatured form, gelatin, as a trigger of the α-Gal syndrome is still controversially discussed (96). The detection of α-Gal on collagen pointed to the possibility that IgE antibodies directed against α-Gal might cause allergic reactions to gelatin. However, this has not yet been proven. When oral challenges were performed with α-Gal allergic patients, they tolerated the oral intake of 10 g of gelatin, but developed urticaria and bronchospasm when a gelatin colloid was administered intravenously (96). A case report of a patient with anti-α-Gal IgE antibodies described also reactions to Haribo gummy bears containing gelatin (97). During controlled food challenges with pork kidney or meat, symptoms started 3–5 h after consumption, whereas during challenges with 250 g of gummy bears, only gastrointestinal symptoms (flatulence and abdominal cramps, followed by flushing, tachycardia, and diarrhea) started 11.5 h after eating (97). Due to the enormous delay in the onset of symptoms, which were solely of gastrointestinal nature, it might be speculated that an intolerance to one of the other ingredients in the gummies rather than an allergic reaction to α-Gal caused the described symptoms. Indeed, it is known that people can develop gastrointestinal manifestations after eating sugar-free Haribo gummy bears, which contain large amounts of maltitol.

α-Gal carrying proteins are also present in mammalian milk and it was shown that IgE antibodies of α-Gal allergic patients recognize α-Gal in cow's milk (35, 98). Recently, γ-globulin, lactoferrin and lactoperoxidase were identified as α-Gal-containing milk allergens (99). However, most of the α-Gal allergic patients (between 80 and 90%) do not react to milk or milk products (e.g., cheese) (32). Therefore, the recommendation to avoid dairy products as well as gelatin is only given to <10% of the patients and it is not part of the standard dietary management of α-Gal allergy (32, 33).

## Delayed Allergic Response to α-Gal

Whereas in case of most food allergies, symptoms start within minutes or 1–2 h after ingestion of the offending food (100), one of the most typical characteristics of the α-Gal syndrome is the late onset of the symptoms, usually starting 3–6 h after consumption of red meat (5). *In vitro* stimulation of basophils from α-Gal allergic patients with α-Gal either bound to glycoproteins or glycolipids showed that the basophil response occurs as fast as after stimulation with other food allergens (5, 101). Furthermore, it is also known that intravenous administration of α-Gal containing drugs, such as the anticancer monoclonal antibody Cetuximab, leads to immediate allergic reactions (4). Based on these observations it was concluded that the delayed reaction after meat consumption is neither due to an intrinsic property of the α-Gal moiety nor to a retarded responsiveness of the basophils. Instead, it is more likely that the digestion and absorption of the α-Gal molecules and the time taken by α-Gal molecules from the ingestion until the appearance in the circulation might cause the delay in the reaction.

Allergens are usually proteins and it is known that the process of digestion and absorption of proteins, from the moment of food ingestion in the oral cavity until the appearance of small peptides or amino acids in the blood takes about 1–2 h (102–104). Allergenic proteins are normally not ingested alone, but together with a complex mixture of proteins and other molecules present in a food source. The term food matrix describes the assembly of nutrient and non-nutrient components of foods and their complex physical and chemical interactions. The food matrix affects the release, accessibility, digestibility, and stability of food compounds and it is known to have a critical impact on the availability and stability of food allergens (105–107). For instance, the resistance to proteolysis of some allergens can be increased by the presence of lipids and polysaccharides in a food matrix (108). Nevertheless, although components of the food matrix can influence the digestibility and allergenicity of food proteins (107, 109), they apparently do not cause a delay in the onset of allergic reactions: in most food allergies, symptoms appear within minutes up to 2 h after food intake and this is in accordance with the time it takes for allergenic peptides to reach the IgE antibodies present on mast cells and basophils.

However, the oligosaccharide α-Gal, is known to exist not only bound to proteins, but also as part of glycolipids (110). Different from proteins, the postprandial peak of the digestion products of triglycerides in the blood occurs between 3 and 4 h after the meal (111). It was therefore suggested that α-Gal bound to lipids and their slower digestion and absorption could be the reason for the delayed response in α-Gal allergic patients (112). This is called the glycolipid hypothesis. In a recent publication we were able to provide first evidence for this theory (113). We carried out a simulated gastric and duodenal digestion of beef lipid and protein extracts, and subsequently added the digestion products to a monolayer of the intestinal Caco-2 cell line grown on permeable supports. We then analyzed the ability of α-Gal conjugated protein and lipid digestion products to cross the intestinal cell layer. We saw that α-Gal was only transported through the cell layer when the cells were exposed to the digested lipids, but not after exposure to the digested proteins, suggesting that only α-Gal bound to lipids could cross the intestinal cells. Krstic et al. meanwhile described that α-Gal-glycosylation actually impairs the transport of proteins across the intestinal epithelium (114).

The digestion and absorption of lipids is certainly an intricate process (Figure 2). Dietary lipids, mainly triglycerides, are first broken down into small droplets by the peristaltic movements of the stomach. Bile salts and phospholipids then coat the droplets of hydrophobic molecules and form so called micelles, solubilizing the lipids in the aqueous medium (115). Pancreatic lipase hydrolyzes the triglycerides inside the micelle into free fatty acids, di- and monoglycerides, and they are carried in the micelles to the surface of enterocytes, where they are absorbed. Inside the enterocytes the free fatty acids and monoglycerides are re-esterified into triglycerides. These are packaged together with phospholipids, cholesterol esters, and apolipoprotein B-48 into lipoprotein particles, called chylomicrons (116), which leave the enterocytes by exocytosis, are released into the lymphatic system and enter the bloodstream *via* the thoracic duct ~4 h after the meal (117). α-Gal is predominantly linked to glycosphingolipids, which represent a diverse group of membrane-bound glycolipids with several different biological functions (118, 119). Not much is known about the resistance of α-Gal containing glycosphingolipids to digestive enzymes. Investigations on other glycolipids, like dietary sphingomyelin or plant sphingolipids in rats, had shown that the majority of these glycolipids is hydrolyzed in the lumen of the small intestine but is not absorbed and transported intact to the lymph (120). However, it might well be that food matrices protect α-Gal containing glycolipids from digestion. If α-Gal-containing glycolipids are ingested together with other lipids, they might be incorporated into micelles and might thus not be accessible, aside from pancreatic lipase, to the digestive enzymes present in the intestinal lumen. Instead, they might be transported to the enterocytes, where they could be incorporated into chylomicrons and in this way reach the systemic circulation several hours after ingestion (6, 112, 113, 118). The incorporation of α-Gal-carrying glycolipids into chylomicrons enables the exposure of several α-Gal epitopes on the surface of chylomicrons, which facilitates the cross-linking of basophils or mast cells bound IgE antibodies and leads to the activation and degranulation of these cells. In our experiments we indeed saw that the α-Gal molecules bound to lipids, which were transported through the intestinal cells, were packed into chylomicrons and that these chylomicrons could *in vitro* activate basophils of an α-Gal allergic individual (113).

Schnabl et al. studied the *in vitro* uptake of another glycosphingolipid, the ganglioside GD3, by human intestinal cells. Interestingly, they observed that, at lower concentrations, most GD3 was metabolized by the enterocytes. However, if higher concentrations of GD3 were added, the additional GD3 was transferred across the intestinal cells (121). Such data are in accordance with the finding that the postprandial increase in chylomicron production depends on the amount of dietary fat ingested in a meal (122). They might also provide an explanation for the observation that α-Gal allergic patients manifest symptoms after consumption of fattier meats or containing higher amounts of glycolipids, such as innards, whereas they better tolerate leaner meats, like venison (33).

The fact that α-Gal bound to glycolipids can induce allergic reactions has further implications. Alcohol and exercise have both shown to increase postprandial lipemia (123, 124), and then also blood levels of α-Gal-carrying glycolipids might be increased. This can explain why patients experience more severe allergic episodes when red meat intake is accompanied by alcohol consumption or followed by exercise (39). On the other hand, the finding that intact glycans bound to proteins are not able to cross the intestinal epithelium (113) might also explain, why IgE antibodies directed to CCDs lack the ability to induce allergic reactions. CCDs exist linked to glycoproteins, but not to glycolipids. Therefore, it might be envisaged that after ingestion of fruits or vegetables containing CCDs linked to proteins, the intact carbohydrates are not able to cross epithelial barriers and can thus not trigger allergic reactions *in vivo*.

The potential of α-Gal to exist as a glycolipid antigen might also be of relevance for the recently described association between anti-α-Gal IgE antibodies and coronary artery disease (125). Among patients with coronary catheterization, those sensitized to α-Gal had an increased burden of atherosclerotic plaques (125). Even though these observations still need to be confirmed, it can be speculated that α-Gal-containing glycolipids from ingested mammalian meat products that are transported to the circulation might activate mast cells bearing α-Gal specific IgEs. The induced release of mediators from the mast cells could contribute to the inflammatory reaction in coronary artery disease (125). In addition, it could well be that *via* α-Gal carrying glycolipids macrophages might also contribute to the development of atheromatous plaques (125). In fact, we observed that anti-α-Gal IgG1 titers were also elevated in red meat-allergic patients (63). The binding of these IgG1 antibodies to α-Gal molecules on the surface of chylomicrons could lead to the recruitment of macrophages and mediate the subsequent phagocytosis of the α-Gal-coated chylomicrons by macrophages, generating foam cells and thus, promoting the formation of atheromatous plaques. However, these hypotheses still need to be further investigated.

## Sensitization to α-Gal

The most remarkable characteristic of α-Gal allergy is that sensitization to the carbohydrate occurs through repeated tick bites, whereas the actual symptoms are triggered by a later exposure to the allergenic molecule by the consumption of mammalian meat (7). Such a course of allergic disease is certainly unique and is also in contrast to allergic reactions caused by other arthropods, like insects, which usually induce immediate cutaneous reactions at the bite site. Although it has been suggested that components present in the insect saliva have immunomodulatory properties, which promote the polarization of naïve T cells toward a Th2 phenotype causing the reactions (126), the majority of individuals develop only transient localized IgE-mediated hypersensitivity reactions to the bites of blood-feeding insects (88).

In contrast, the ability of humans to develop hypersensitivity reactions after repeated tick bites resembles the mechanism of acquired tick resistance (ATR) observed in several animal species (8). Thus, it was suggested that the α-Gal-specific IgE response in humans is an evolutionary adaptation associated with ATR and allergic klendusity with the trade-off of developing α-Gal syndrome. Allergic klendusity refers to a disease-escaping ability produced by the development of hypersensitivity to an allergen (8). Bell et al. (127) proposed for the first time that allergic klendusity was the immune property by which tick-sensitized rabbits developed resistance to tick-borne *Francisella tularensis* infection.

To elucidate the mechanisms behind the unusual way of sensitization in case of the α-Gal syndrome, specific research focused on the analysis of the tick saliva and on discovering the source of α-Gal present in the saliva. Even though Hamsten et al. (128) were able to detect α-Gal epitopes in the gastrointestinal tract of *Ixodes ricinus* ticks, it was still a matter of debate whether these α-Gal molecules were synthesized by the tick or had their origin in a previous blood meal of the tick, or were produced by symbionts or parasites present in the tick. Fischer et al. (129), however, detected the presence of α-Gal epitopes in the midgut, hemolymph and salivary glands of *I. ricinus* females regardless of their feeding status. Meanwhile, three genes involved in the synthesis of α-Gal have been identified in *I. scapularis* genome (80) and the presence of α-Gal has also been confirmed in *Amblyomma americanum* ticks fed on human blood, which lacks α-Gal (130). All these findings support the view that α-Gal moieties present in tick tissues and saliva do not originate from a prior blood meal but are produced by the ticks themselves.

**Figure 3:**
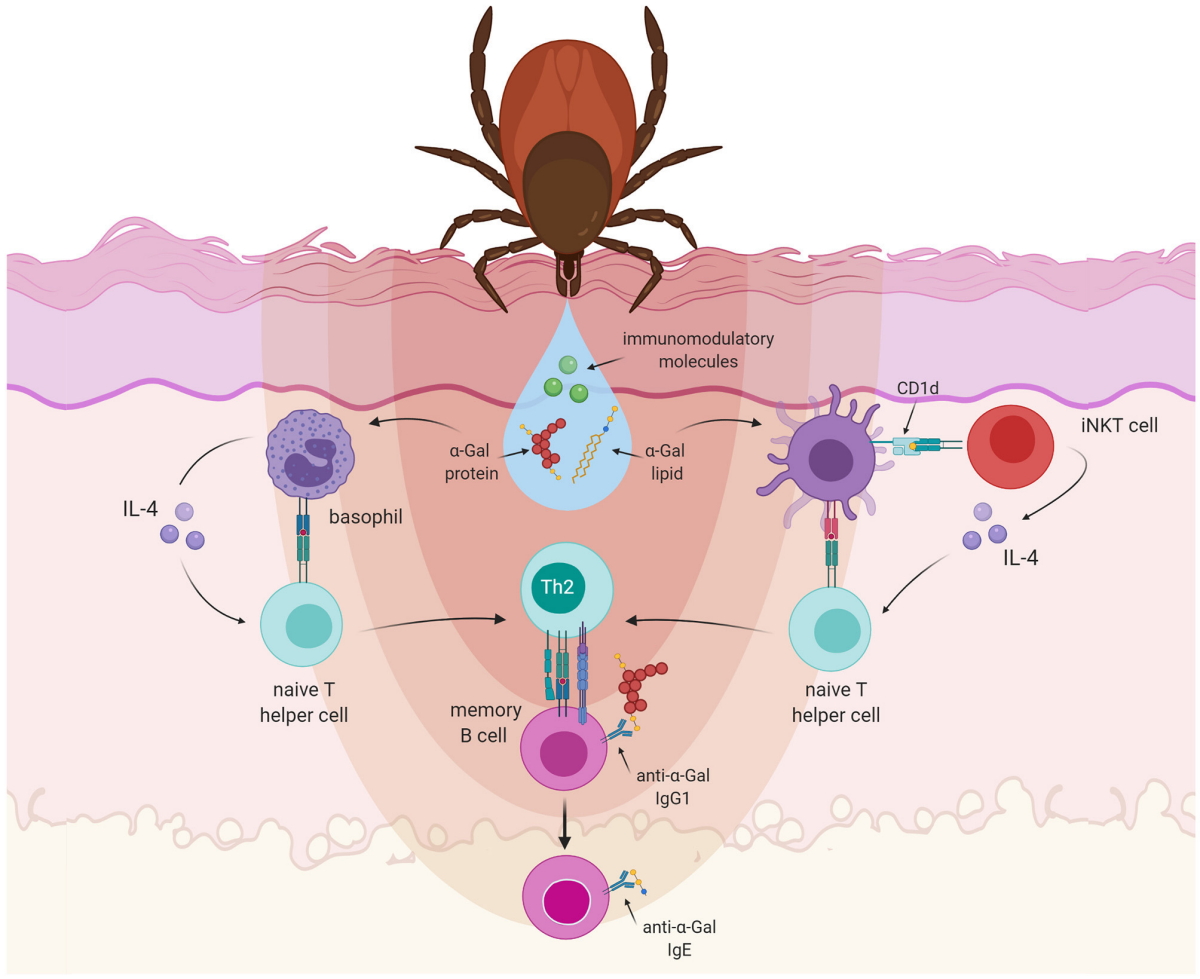
Allergic sensitization to α-Gal. Tick saliva contains immunomodulatory molecules together with α-Gal carrying proteins or lipids. Basophils are recruited to the tick-feeding sites and accumulate in the skin after a repetitive tick infestation. Basophils can act as non-professional antigen presenting cells, capable of secreting IL-4. Lipids containing α-Gal could also be presented to iNKT cells in the context of CD1d, which are known to produce IL-4 abundantly. IL-4 will skew the differentiation of naT cells recognizing tick saliva antigens into type 2 T helper (Th2) cells. Th2 cells will subsequently interact with anti-α-Gal-specific B cells and induce class-switch recombination leading to the production of IgE antibodies.

To investigate how tick bites can initiate the anti-α-Gal IgE response, Choudhary et al. made use of an α-Gal knockout mouse model. They saw that sensitization with tick salivary gland extract caused the generation of IgE antibodies to α-Gal and the development of allergic reactions to mammalian meat (131). These experiments illustrate the important role of the tick saliva for the development of α-Gal allergy. The saliva of ticks is a complex mixture of substances, several of them also with immunomodulatory properties. With the injury caused by the tick mouthparts that disrupt the epidermis and enter the dermis of the host skin, the mechanisms of wound healing begin in the host (132): coagulation, vasoconstriction, and platelet aggregation are followed by responses of the innate and adaptive immune system (133). For an effective blood feeding, the tick must be able to counteract the defense mechanisms of the host. Indeed, bioactive molecules present in the tick saliva can suppress the host's hemostatic as well as immune responses that impede efficient feeding (134) and that might damage the tick (135). Tick saliva has been shown to decrease the production of the proinflammatory mediators IL-12, IL-1β or TNF-α (132), while promoting an increased expression of anti-inflammatory cytokines like TGF-β or IL-10 (136). These effects might be mediated by molecules such as Prostaglandin E2 (PGE2), which is very abundant in tick saliva. PGE2 induces vasodilation and impairs wound healing while reducing inflammation (137). Furthermore, in mice infested by ticks high TGF-β levels have been observed, together with increasing amounts of IL-10 and IL-4 after every exposure to ticks (136). This suggests that repeated exposure to tick saliva could skew the polarization of the immune response toward a Th2 profile, which induces the development of allergies and suppresses a pro-inflammatory Th1 response (135) (Figure 3).

Another issue of importance is the role of the skin in the sensitization process. It has been suggested that the skin might represent an important site of sensitization to food allergens (138, 139). Atopic dermatitis has been shown to be strongly associated with food allergies (140) and many patients suffering from allergy to peanuts or tree nuts experienced their first allergic reaction the first time they consumed the foods. It was thus concluded that allergic sensitization had to occur already before by a different route, for instance by dermal exposure (141). It could well be that the injury caused in the skin by tick bites might facilitate the sensitization process to α-Gal. In this context, it has been proposed that basophils, acting as non-professional antigen presenting cells and producing IL-4, may play a role in allergic sensitization and the initiation of Th2 immune responses, inducing the differentiation of naive CD4^+^ T cells into Th2 cells (142, 143). It is known that basophils are recruited to tick-feeding sites, where they accumulate in the skin and might be involved in IgE antibody-mediated acquired tick resistance (144) (Figure 3). Based on these findings it might be envisaged that the exposure to gut microbes leads to basal anti-α-Gal IgG levels, whereas later exposure to α-Gal *via* tick bites in the context of basophil-derived IL-4 might induce further class-switch recombination in anti-α-Gal memory B cell clones, leading to the production of IgE antibodies (145). However, this hypothesis is discussed controversially, because basophils do not seem to play a role in promoting T cell proliferation in allergic immune response in humans (146).

Keeping in mind that the α-Gal epitope can also be part of glycolipids, it might be speculated that α-Gal-carrying lipids can also be present in the tick saliva. In this case a subset of T cells, the innate-like, invariant natural killer T cells (iNKT) might contribute to the process of sensitization to α-Gal. iNKT cells recognize lipids presented to them by CD1d molecules (non-polymorphic, MHC class I-like proteins) and they are able to produce IL-4 (147) (Figure 3). It has been observed that the median frequency of circulating activated CD69^+^ iNKT cells in α-Gal allergic patients was 2.5-fold higher than in control subjects, indicating a trend toward a higher frequency of circulating iNKT cells in individuals allergic to α-Gal. Furthermore, the expression patterns for different genes associated with iNKT cell development and effector functions appeared to be also different in the α-Gal allergic patients and the control group (148).

Besides ticks, it has been suggested that other members of the Arachnida class, Trombiculidae, also commonly known as “chiggers,” may contribute as well to the allergic sensitization to α-Gal (149). Certain individuals who were diagnosed with the α-Gal syndrome, reported chiggers, but not tick bites, in the weeks prior their first allergic episode after eating red meat. Often, these subjects had been bitten by ticks long before (149). Since sensitization to α-Gal seems to require repeated tick bites (7), the exposure to bites from chiggers after a previous tick bite may be behind the development of the α-Gal syndrome in these patients. Interestingly, 5.5% of α-Gal IgE-sensitized individuals that answered a questionnaire at the University of Virginia, reported a history of chigger bites, but no bites from ticks (149). However, it is not known yet whether the α-Gal epitope is even present in the saliva or gastrointestinal tract of Trombiculidae.

## Open Questions

Allergy to α-Gal is certainly an uncommon form of allergy and, even though our knowledge about the disease has increased significantly over the last 10 years, many questions still need to be answered. One of the most intriguing ones is still how and why an allergic sensitization to α-Gal is initiated after a tick bite. Owing to the presence of immunomodulatory molecules in the tick saliva, one would expect to see allergic reactions to it. Instead, even though cases have been reported in the United States and in Europe, allergy to ticks or tick saliva is only common in tick hyper-endemic areas in Australia (150–152) and only recently some of the proteins associated with allergic reactions to tick bites have been characterized (152). To understand why IgE antibodies are raised against α-Gal and not against any of the proteins present in the saliva and to reveal the role of the skin would undoubtedly contribute to a better knowledge about the sensitization process. Considering that the skin is suggested as an important site of allergic sensitization to foods (138, 139), understanding the course of the sensitization to α-Gal after a tick bite could serve as a model to understand the sensitization process also in other food allergies.

It would further be of importance to determine the specific role of the intestinal microbiome in the development of α-Gal allergy. Individuals with anti-α-Gal IgE antibodies appear to have also higher titers of IgG1 and IgG2 antibodies directed against α-Gal (63). It is not known whether these individuals presented already higher levels of IgG antibodies to α-Gal prior to the development of α-Gal allergy, or whether the increase in IgG antibody levels was induced by the tick bites. If elevated anti-α-Gal IgG1 and IgG2 levels precede tick infestation, it will be of interest to identify bacteria of the intestinal microbiome that have an immunomodulatory effect and influence the anti-α-Gal antibody titers.

Although we showed in an *in vitro* approach that α-Gal is transported across intestinal cells only when the molecule is bound to lipids and in this way provided the first evidence that the binding of α-Gal to lipids might cause the delay in the allergic responses to mammalian meat, details about the mechanism of the delayed allergic response are still missing. In this context, it would be important to know the exact nature of α-Gal-carrying lipids present in mammalian meat, the modifications they undergo during digestion and absorption and the nature of the α-Gal-containing lipid that leaves the enterocytes packed into chylomicrons.

From a clinical point of view, a pivotal question is why only a small proportion of α-Gal-sensitized individuals actually develop a clinically manifest α-Gal syndrome, and by which diagnostic procedures these individuals at risk might be reliably identified. In view of the increasing use of multiplex assays for allergy screening, the large number of clinically irrelevant α-Gal sensitizations to be expected represents a considerable diagnostic challenge for clinicians.

## Author Contributions

PR-C and IS conceived the study and drafted the manuscript. WH, AC-C, AH, and JF wrote specific parts of the review. PR-C prepared the figures. All authors reviewed and approved the manuscript in its current form.

## Funding

The preparation of this manuscript was supported by research grant P33867 of the Austrian Science Fund (FWF).

## Conflict of Interest

The authors declare that the research was conducted in the absence of any commercial or financial relationships that could be construed as a potential conflict of interest.

## Publisher's Note

All claims expressed in this article are solely those of the authors and do not necessarily represent those of their affiliated organizations, or those of the publisher, the editors and the reviewers. Any product that may be evaluated in this article, or claim that may be made by its manufacturer, is not guaranteed or endorsed by the publisher.
